# Inadequate Anti–Factor Xa Levels With Daily 40-mg Enoxaparin After Cardiac Surgery

**DOI:** 10.1016/j.atssr.2023.12.024

**Published:** 2024-02-08

**Authors:** Hyungjoo Kim, Joshua Newman, Hugh Cassiere, Alan Hartman, Pey-Jen Yu

**Affiliations:** 1 Department of Cardiovascular and Thoracic Surgery, Zucker School of Medicine at Hofstra/Northwell, Manhasset, New York

## Abstract

**Background:**

Cardiac surgery patients are at increased risk for venous thromboembolism (VTE). Prevention is the most critical strategy to reduce VTE-associated morbidity and death. However, there is a lack of data on the optimal approach to VTE prophylaxis in this population of high-risk patients. This study aimed to assess whether the standard dose of enoxaparin, the subcutaneous injection of 40 mg of enoxaparin daily, achieves adequate anti–factor Xa (aFXa) levels for VTE prophylaxis in patients after open heart surgery.

**Methods:**

All patients with open heart surgery with cardiopulmonary bypass from August to December 2022 who received at least 3 consecutive doses of subcutaneously administered enoxaparin were included in the study. Patients receiving therapeutic anticoagulation, patients who underwent cardiac transplantation or placement of ventricular assist device, and patients with renal insufficiency were excluded. Serum aFXa was measured 0.5 to 1 hour before the fourth dose to attain the steady-state trough levels.

**Results:**

Data were completed for 44 patients. The target aFXa level was between 0.10 and 0.20 IU/mL for the avoidance of both underanticoagulation (≤0.10 IU/mL) and overanticoagulation (>0.20 IU/mL). The mean was 0.049 IU/mL with SD of 0.026 IU/mL, which was statistically significantly lower than the lower end of the target aFXa values (0.10 IU/mL; *t*_43_ = −13; *P* < .001; *d* = −1.9; 99% CI, −0.059 to −0.043).

**Conclusions:**

The daily subcutaneous administration of 40 mg of enoxaparin leads to subprophylactic aFXa levels for most patients who undergo cardiac surgery. Further studies on the clinical relevance are warranted.

**In Short**

▪ Enoxaparin is frequently used for prophylaxis of venous thromboembolism after cardiac operations; however, there is a lack of consensus on adequate enoxaparin dosing.
▪ In the study of cardiac surgery patients, it was found that of 44 patients who received the standardized enoxaparin dose for 3 consecutive days until the steady state was achieved, 43 expressed subprophylactic steady-state trough anti–factor Xa levels, indicating that the current standardized venous thromboembolism regimen may not be adequate for this population of patients.

Venous thromboembolism (VTE), including deep venous thrombosis (DVT) and pulmonary embolism (PE), is a critical patient safety issue for cardiac surgery patients. Studies report that silent DVT develops in 13% to 17.4% of patients after cardiac surgery despite thromboprophylaxis.^1^ A meta-analysis on VTE after cardiac surgery found the median incidence of symptomatic DVT, PE, and fatal PE to be 3.2%, 0.6%, and 0.3%, respectively.^1^ DVT and PE have been associated with increased mortality after cardiac surgery, with approximately 10% to 20% of unexplained deaths after cardiac surgery attributable to fatal PE.^1^ Cardiac surgery patients have significant risk factors for VTE, which include advanced age, need for extensive operations, previous VTE, obesity, left or right ventricular heart failure, use of central venous catheter, and other baseline cardiac and thoracic conditions.^1^ Because prevention is proposed as the essential approach to prevent VTE-associated morbidity and mortality, the American College of Chest Physicians clinical practice guideline recommended VTE prophylaxis with low-dose unfractionated heparin or low-molecular-weight heparin (LMWH) for high-risk patients undergoing cardiac surgery.^2^ The National Institute for Health and Care Excellence explicitly recommends use of LMWH for most patients undergoing cardiac surgery.^3^

Enoxaparin is an LMWH that is frequently used for VTE, and the anti–factor Xa (aFXa) level can be used as a marker for enoxaparin activity.^4^ Based on trauma and surgical literature, steady-state aFXa trough levels >0.10 IU/mL and ≤0.20 IU/mL have been suggested to provide adequate VTE prophylaxis without an increased risk of bleeding.^4^, ^5^, ^6^ Although enoxaparin 40 mg once daily is frequently used for VTE prophylaxis for cardiac surgery patients,^1^ there is no evidence that such a dose is adequate for this population of patients. This study assesses whether the standard enoxaparin VTE prophylaxis dose achieves adequate steady-state trough aFXa levels for cardiac surgery patients.

## Patients and Methods

Patients undergoing cardiac operations at a single quaternary academic center with use of cardiopulmonary bypass from August to December 2022 who received at least 3 consecutive doses of subcutaneously administered enoxaparin prophylaxis were eligible for inclusion. The following patients were excluded: patients receiving therapeutic anticoagulation, patients who underwent cardiac transplantation or placement of ventricular assist device, patients on extracorporeal membrane oxygenator support, and patients with renal insufficiency. The study was conducted as a quality improvement project for the cardiothoracic surgery department at the institution, and institutional review board approval for the human subject research was waived.

Patients were started on a prophylactic dose of enoxaparin 1 day after the surgery on the basis of the institution’s standardized VTE regimen: for patients with body mass index (BMI) <40 kg/m^2^, patients receive 40 mg of subcutaneously administered enoxaparin per day; and for patients with BMI >40 kg/m^2^, patients receive 30 mg of subcutaneously administered enoxaparin twice daily. Serum aFXa was measured 0.5 to 1 hour before the fourth dose to attain the steady-state trough levels.^4^, ^5^, ^6^ Plasma samples were analyzed by the Liquid Anti-Xa kit (Werfen Inc) through an ACL TOP 750 instrument calibrated for LMWH to evaluate the anti-Xa activity.

Data were analyzed with SPSS version 29 statistical software (SPSS Inc). Based on the existing literature,^4^, ^5^, ^6^ the target aFXa value for VTE prophylaxis was >0.10 IU/mL and ≤0.20 IU/mL, to which the mean was compared by a 1-tailed *t*-test.

## Results

Data were completed for 44 patients. All these patients had a BMI <40 kg/m^2^ and received 40 mg of subcutaneously administered enoxaparin daily for at least 3 days after surgery. The target aFXa level was between 0.10 and 0.20 IU/mL for the avoidance of both underanticoagulation (≤0.10 IU/mL) and overanticoagulation (>0.20 IU/mL). Only 1 of the 44 patients had the prophylactic steady-state trough aFXa level (0.12 IU/mL) after subcutaneous injection of 40 mg of enoxaparin once daily (Figure). The mean aFXa level of the patients given the standardized dose of enoxaparin was 0.049 IU/mL, with SD of 0.026 IU/mL. This was statistically significantly lower than the lower end (0.10 IU/mL) of the target aFXa values (*t*_43_ = −13; *P* < .001; *d* = −1.9; 99% CI, −0.059 to −0.043), indicating that patients given the prophylactic dose of enoxaparin do not reach the target aFXa levels.

**Figure:**
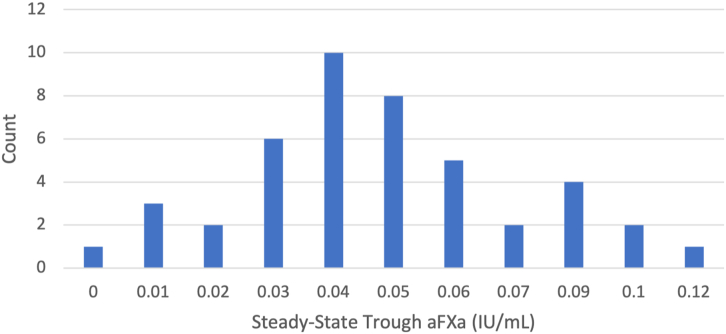
The distribution of steady-state trough anti–factor Xa (aFXa) levels for cardiac surgery patients who received subcutaneous injection of 40 mg of enoxaparin daily for at least 3 consecutive days (n = 44).

The data recruitment ended early, given the consistently low aFXa levels between patients.

## Comment

Prophylactic enoxaparin surveillance may be based on aFXa peak or trough levels. Malinoski and coworkers^6^ conducted an enoxaparin study of 54 critically ill surgical and trauma patients. They found that 50% of the patients had low steady-state trough aFXa levels (≤0.10 IU/mL), and 37% of those with subprophylactic levels had VTE. They also found no difference in peak aFXa levels between those who had VTE and those who did not. A study by Ko and associates^4^ comparing trauma patients who received standardized VTE prophylactic dosage of enoxaparin vs patients who had their dosage of enoxaparin adjusted to maintain trough levels >0.10 IU/mL found that the incidence of VTE was almost 7-fold higher in patients receiving the standard vs adjusted doses of enoxaparin (7.6% vs 1.1%). As the stated literature supported a correlation between trough aFXa levels and DVT events, we chose to monitor trough enoxaparin activity rather than peak levels for this study.

Our study suggests that standard enoxaparin dosing may not be sufficient to achieve the target steady-state trough aFXa levels for VTE prophylaxis in cardiac surgery patients. However, subcutaneous administration of enoxaparin has been shown to result in subprophylactic aFXa levels in critically ill, trauma, and other surgical patients.^4^, ^5^, ^6^ Several factors are associated with unpredictable responses after standard enoxaparin dosing, such as weight, creatinine clearance, peripheral edema, multiple organ dysfunction, and use of vasoconstrictors like norepinephrine.^4^^,^^7^^,^^8^ Dörffler-Melly and colleagues^7^ found that use of norepinephrine led to lower aFXa levels compared with patients without the administration of norepinephrine. Ko and associates^4^ suggested that higher creatinine clearance may be associated with subprophylactic aFXa levels in trauma patients. Haas and coworkers^8^ demonstrated that peripheral edema and weight are associated with decreased aFXa levels in critically ill patients after enoxaparin administration. As cardiac surgery patients are frequently receiving some vasopressor support and have some degree of renal dysfunction and edema in the immediate postoperative period, it is not surprising that their aFXa levels with standard enoxaparin dosing are not adequate. Although 1 of the original objectives of this study was to assess risk factors in cardiac surgery patients for subprophylactic enoxaparin response, it was determined that a subgroup analysis would not be feasible, given that 43 of the 44 patients fell into the subprophylactic range of aFXa.

Cardiac surgery patients may also be more resistant to standard doses of enoxaparin as consistently low antithrombin III (AT III) levels have been shown after cardiac surgery with cardiopulmonary bypass.^9^ Enoxaparin works by binding to AT III to inactivate clotting factors, preferentially factor Xa, thereby blocking the conversion of prothrombin to thrombin, which is the coagulation cascade’s final common pathway. Inherently low AT III after cardiopulmonary bypass may attenuate the enoxaparin effect; however, randomized controlled trials aimed at increasing AT III levels with post–cardiac surgery AT III supplementation failed to demonstrate any VTE prophylaxis benefit and did not increase AT III levels.^10^ The relationship between low AT III in cardiac surgery patients and subprophylactic aFXa and the underlying mechanisms remain unclear.

There are several limitations to our study. Our result demonstrates the enoxaparin response from a pharmacodynamic perspective; further studies will be needed to assess the clinical relevance of low aFXa trough levels. As mentioned, the data entry ended early and provided a small sample size. A larger sample population with an evaluation of VTE events would be needed to analyze individual risk factors and to offer more robust conclusions. The confounding variables that affect the enoxaparin response, such as the use of vasopressors and peripheral edema, need to be assessed with a larger sample size and downstream VTE events. Patients were recruited at a single center, so the study’s generalizability may be limited.

In conclusion, the current VTE prophylaxis regimen leads to suboptimal aFXa levels for most patients who undergo cardiac surgery. Future studies are warranted to evaluate the clinical significance of inadequate aFXa levels in this population of patients.
